# Serum Sortilin Levels as a Biomarker for Metabolic and Hormonal Dysregulation in Polycystic Ovary Syndrome

**DOI:** 10.3390/jpm15020070

**Published:** 2025-02-15

**Authors:** Pinar Alarslan, Mehmet Doruk

**Affiliations:** 1 Department of Endocrine and Metabolic Diseases, Istanbul Aydin University, Medical Park Florya Hospital, Besyol, Florya, Akasya Sk., 34295 Kucukcekmece/Istanbul, Turkey; 2 Department of Endocrine and Metabolic Diseases, İzmir Bozkaya Research and Education Hospital, Bahar, Saim Çıkrıkçı Cd., 35170 Karabağlar/İzmir, Turkey; drmehmetcalan@gmail.com

**Keywords:** sortilin, LDL-C, lipid metabolism, insulin resistance

## Abstract

**Background/Objectives**: Polycystic ovarian syndrome (PCOS) is a complex endocrine disorder affecting up to 15% of reproductive-age women, characterized by hyperandrogenism, chronic oligo-ovulation, and metabolic dysfunction. This study aims to evaluate serum sortilin levels in women with PCOS for the first time and investigate their potential associations with metabolic and hormonal alterations. **Material and Methods:** Eighty PCOS patients and 80 healthy controls were included; serum sortilin levels were measured using ELISA kits, with documented intra-assay and inter-assay variations below 6% and 8%, respectively, ensuring high specificity and sensitivity. **Results:** Serum sortilin levels were significantly elevated in PCOS patients (69.51 ± 27.75 pg/mL) versus controls (48.60 ± 21.20 pg/mL) (*p* < 0.001). PCOS patients exhibited higher mean HOMA-IR, free androgen index values, serum glucose, insulin, triglycerides, high-sensitivity C-reactive protein, luteinizing hormone, total testosterone, and DHEA-S levels, alongside reduced high-density lipoprotein cholesterol and sex hormone-binding globulin levels (all, *p* < 0.05). Notably, inverse correlations were observed between sortilin and low-density lipoprotein cholesterol levels in both groups (*p* = 0.028 and 0.033). **Conclusions:** This pioneering study indicates that serum sortilin may be implicated in PCOS pathogenesis and serves as a potential biomarker for metabolic dysfunction in PCOS. Larger, diverse studies with longitudinal designs are needed for further validation.

## 1. Introduction

Polycystic ovarian syndrome (PCOS) is a complex endocrine and metabolic disorder affecting up to 15% of reproductive-age women that leads to hyperandrogenism, chronic oligo-ovulation, or anovulation [1]. We hypothesize that elevated sortilin levels may represent a novel biomarker involved in the pathophysiology of PCOS by modulating lipid and glucose metabolism. However, the potential influence of PCOS subtypes (e.g., based on phenotypic variations) on biochemical markers should be considered. The clinical heterogeneity of the disease seems to be adjusted by various factors, including genetic susceptibility; intrauterine conditions; epigenetic mechanisms; and environmental factors, such as nutrition, body weight, and lifestyle [2]. It is now clearly known that PCOS is associated with metabolic co-morbidities ranging from obesity, dyslipidemia, and insulin resistance (type 2 diabetes mellitus) to hypertension and cardiovascular disorders. However, the underlying mechanisms linking PCOS to these disorders remain as poorly understood topics [3]. The identification of novel metabolic factors contributing to the pathogenesis of PCOS provides additional data to better understand the disease.

Sortilin, a member of the mammalian vacuolar protein sorting 10p domain (VPS10) protein family, is a transmembrane glycoprotein that has been understood to be a novel mediator for lipids, cytokines, and enzymes that serve in the regulation of intracellular protein transportation [4]. It is also functionally related to various biological processes, including the regulation of gene expression, ossification, cell death, neuronal and tumor cell survival, as well as glucose and lipoprotein metabolism [5].

Sortilin, known as a novel mediator in lipid metabolism and glucose regulation, has been implicated in cardiovascular and metabolic diseases, warranting further investigation into its potential impact on metabolic dysfunction in PCOS. Despite being a membrane-bound protein, small amounts of sortilin have been demonstrated in the bloodstream [6]. Recent studies have suggested crucial roles for sortilin in neurological, cardiovascular, and metabolic diseases such as type 2 diabetes mellitus, and it has been associated with dysregulation in lipoprotein metabolism [7]. However, no studies to date have evaluated sortilin levels in patients with PCOS, highlighting a research gap that this study aims to address.

Serum sortilin levels were measured for the first time in women diagnosed with Polycystic Ovarian Syndrome (PCOS), and their potential associations with key clinical and metabolic features of PCOS were investigated. This study hypothesizes that serum sortilin levels may be elevated in PCOS patients compared to healthy controls and may exhibit significant associations with metabolic and hormonal alterations characteristic of the syndrome. The primary aim of this study is to evaluate serum sortilin levels in women with PCOS for the first time and investigate their potential associations with metabolic and hormonal dysregulation characteristic of the syndrome. There are strong expectations that the levels of sortilin will adopt considerable associations with those altered metabolism and hormonal changes of PCOS.

## 2. Materials and Methods

The study, which was a case-control study, was conducted from April 2021 and July 2021 in Istanbul Aydin University Medicalpark Florya. The study included 80 women diagnosed with PCOS based on the Rotterdam Consensus Criteria, requiring at least two of the following: oligo-ovulation or anovulation, clinical or biochemical hyperandrogenism, and polycystic ovarian morphology on ultrasound [8]. Participants were aged 18–40 years, had a BMI within a defined range, and had not used medications affecting glucose or lipid metabolism in the past six months. Patients with significant metabolic, endocrine, cardiovascular, autoimmune, or inflammatory diseases, as well as those who were pregnant, lactating, or had a history of smoking or alcohol abuse, were excluded. All participants provided written informed consent. According to the patients’ reports, they had not taken medication for over six months that affected glucose or lipid metabolism. In an effort to minimize any potential selection bias, recruitment procedures were carried out in a controlled setting.

Biochemical hyperandrogenism was evidently displayed by testosterone and/or dehydroepiandrosterone sulfate (DHEA-S) levels above specified clinical levels as prescribed. For hyperandrogenism, a free androgen index (FAI) greater than or equal to 5% was also used. Hirsutism was evaluated by the use of a modified Ferriman–Gallwey (FG) score in which hair growth in 11 androgen-dependent sites is assessed. A total FG score of 8 or greater is regarded as a diagnosis of hirsutism. FG scoring was carried out in a standardized manner and only recruited females aged 12 and above.

We did not include participants with active hyperthyroid or hyperprolactinemia disorders; women who were lactating or pregnant; and participants suffering from diseases that have a systemic effect on insulin sensitivity such as impaired glucose tolerance, diabetes, or gestational diabetes. Moreover, participants having autoimmune diseases, having chronic inflammatory diseases, having hypertension, having a history of coronary artery disease (CAD), having a history of chronic hyperlipidemic episodes, suffering from congestive heart failure, experiencing chronic or acute infections, and having a chronic history of smoking or alcohol abuse were also excluded. Patients who had been treated for conditions that might affect insulin sensitivity, serum lipid concentration, steroid concentration, the distribution of fat in the body, or any of these within six months of recruitment were not included in the study population.

During clinical assessments, patients were thoroughly selected, including obtaining detailed medical histories and measurements of height, weight, and waist circumference for each patient. BMI is one of the ways that allows for quick assessments of overweight and obese people and has a formula weight (kilograms)/height (meters) squared. A trained physician measured blood pressure while the patient was seated comfortably and undisturbed for 15 min. All ultrasonographic measurements were carried out by one investigator at the highest magnification so that there wass uniformity across all participants.

The study protocol was reviewed and approved by the Non-Interventional Clinical Research Ethics Committee of Istanbul Aydin University (No: 2021/446, Date: 11 April 2021). Women willing to participate provided both verbal and written informed consent before inclusion in the study.

### 2.1. Biochemical Analysis

Blood samples were collected from the antecubital vein after a fasting period of overnight duration, during the early follicular phase (on 3–5 days of the natural menstrual cycle) or during progesterone-withdrawal bleeding if the patient was amenorrheic.

Total cholesterol, high-density lipoprotein cholesterol, triglycerides (TG), C-reactive protein, and glucose compact test (serum glucose and hemoglobin a1c, (HbA1c)) were evaluated utilizing colorimetric methods via the Olympus AU 2700 autoanalyzer (Beckman Coulter Inc., Brea, CA, USA). LDL-C was determined using the Friedewald equation. All subjects fasted overnight, a glucose load of 75 g OGTT was undertaken, and blood glucose levels were taken at 0 and 2 h post lactogenic intake.

Serum FSH, luteinizing hormone, estradiol, and insulin were assessed on the UniCel DxI 800 (Beckman Coulter Inc., Brea, CA, USA) utilizing chemiluminescent enzyme immunoassay. The insulin resistance (IR) was defined through a homeostasis model assessment of IR by fasting blood glucose (mg/dL) × fasting insulin (mIU/L)/405.

Total testosterone, DHEA-S, and sex hormone-binding globulin (SHBG) levels were determined using a passive immunoassay method with a UniCel DxI 800 (Beckman Coulter Inc., Brea, CA, USA). FAI is an acronym that stands for the free androgen index, which is equal to total testosterone/SHBG × 100.

Serum sortilin levels were measured with an ELISA Kit, which is commercially available (Elabscience Ltd., Wuhan, China) and accords to the supplier’s stipulations. The assay measurement range was 9.17–126.51 pg/mL, with intra-assay and inter-assay coefficients of variation of less than 6% and 8%, respectively.

### 2.2. Statistical Analysis

All the statistical analysis was carried out using the Microsoft application, SPSS v18.0 (SPSS Inc., Chicago, IL, USA). The normality of the data was verified using the Kolmogorov–Smirnov test. The continuous variables were expressed as the mean with the respective standard deviation (SD). In the case of normally distributed variables, the independent samples t-test was used to compare them. The relationships between continuous variables were assessed by using Pearson’s correlation coefficients. Results with *p*-values of less than 0.05 were considered statistically significant.

## 3. Results

Within the given study, 80 patients who were diagnosed with PCOS and 80 healthy participants were analyzed. It was found that the mean age for patients who were diagnosed with PCOS was approximately 30.05 ± 6.85 years, while that for controls was 30.12 ± 6.49 years (*p* = 0.945). The two groups did not appear to differ significantly with regard to anthropometric indices or clinical features such as body mass index, waist circumference, and systolic and diastolic blood pressure measurements (all, *p* > 0.05) (Table 1).

The mean values were observed to be greater in serum sortilin levels of the PCOS cohort (69.51 ± 27.75 pg/mL) in comparison to controls (48.60 ± 21.20 pg/mL) (*p* < 0.001). The mean values of glucose, insulin, HOMA-IR, TG, hs-CRP, LH, total testosterone, FAI, and DHEA-S were noted to be higher among subjects in the PCOS group (all, *p* <0.05); however, the mean levels for HDL-C and SHBG of PCOS patients were significantly lower when compared with healthy subjects (all, *p* < 0.05). There were no significant differences in the levels of HbA1c, total cholesterol, LDL-C, estradiol, and FSH between the two groups (all, *p* > 0.05). The clinical characteristics of the subjects are outlined in Table 1.

The sortilin concentration was observed in relation to the clinical and biochemical characteristics of the participants; the findings are illustrated in Table 2. Sortilin levels correlated positively with LDL-C levels only in the control group, while an inversed correlation was found in the patients with PCOS and the control group (*p* = 0.028 and 0.033, respectively). Other variables that had no significant correlations with sortilin concentration were age, BMI, waist circumference, and blood pressure values, as well as serum insulin, HOMA-IR, HbA1c, FAI, and Hs-CRP in both the patients with PCOS and controls (all *p* > 0.05).

## 4. Discussion

Our results demonstrate increased serum sortilin levels in patients diagnosed with PCOS who also had higher glucose, insulin, HOMA-IR, TG, Hs-CRP, LH, total testosterone, FAI, and DHEA-S levels, whereas the serum levels of HDL-C and SHBG were lower. However, despite these associations, the causative role of sortilin remains unclear, and further longitudinal studies are necessary to explore mechanistic pathways. We found that sortilin was negatively correlated with LDL-C in both patients with PCOS and healthy participants. An analysis of relationships showed a lack of significant correlations between sortilin levels and clinical/biochemical characteristics.

PCOS is a common and heterogeneous systemic neuroendocrinopathy causing androgen excess, ovarian dysfunction, and polycystic ovarian morphology [1]. Studies have shown that hyperandrogenism stimulates abdominal obesity in patients with PCOS, leading to dyslipidemia, insulin resistance, and hyperinsulinemia [9]. Insulin resistance may promote ovarian and adrenal hormone production, may augment the frequency of LH release, and may inhibit the production of SHBG, which, in turn, elevates testosterone activity [9,10]. An elevated LH/FSH ratio may also induce androgens and suppress estrogen expression, while elevated androgen and decreased estrogen can also aggravate LH/FSH ratio [11]. As expected, we found increases in total testosterone, LH, DHEA-S, hs-CRP, and FAI values, as well as decreased SHBG levels, in patients diagnosed with PCOS. Our results support the hypothesis that excessive amounts of androgenic hormones, particularly increased circulating testosterone, play a pivotal role in the development of most of the metabolic and reproductive alterations associated with PCOS. These findings align with the recent literature, which suggests that hyperandrogenism is not only a hallmark feature of PCOS but also a key driver in the development of metabolic complications, as highlighted in the literature [12]. The potential molecular mechanisms and novel candidate biomarkers responsible for metabolic dysfunction related to PCOS are still being investigated.

Sortilin is recognized as an important regulatory protein that works as a receptor for neurotrophic factors and neuropeptides. It also serves as a co-receptor for various cytokine receptors, tyrosine receptor kinases, G-protein-coupled receptors, and ion channels. Sortilin is highly expressed in metabolically active tissue such as the liver, brain, skeletal muscles, adipose tissue, and ovaries [13]. For example, sortilin overexpression has been observed in ovarian tumor cells, thereby suggesting that it can be a useful biomarker of ovarian carcinoma [14]. Recent reviews, such as Verma et al., suggest that AI-based diagnostic models may help refine biomarker identification in PCOS, emphasizing the need to integrate sortilin into future computational models for enhanced diagnostic accuracy [15].

Animal model studies, as well as those carried out on cultured cells, ruled out the possibility of alternative explanations and confirmed that sortilin is associated with other biological processes such as glucose and lipid metabolism. Furthermore, it is considered one of the key players in inflammatory processes and metabolic diseases such as type 2 diabetes mellitus and some cardiovascular diseases [7]. Sortilin increases sensitivity to insulin by enhancing the expression of the glucose transporter type 4 (GLUT4) in muscle and fat tissues. In addition, both GLUT4 and sortilin are required for insulin-facilitated glucose transport into cells [16]. Mice with a heterozygous knockout of the GLUT4 gene developed insulin resistance in muscles, which resulted in type 2 diabetes mellitus [17].

Li et al. showed in their mouse study that hepatic sortilin 1 is an insulin target and is especially vulnerable to insulin resistance-induced post translational hepatic degradation [18]. Sortilin could participate in neurotensin receptor mediated protection and the maintenance of pancreatic beta-cells or have pro-apoptotic effects when associated with p75 neurotrophin receptor [19].

Type 2 diabetes mellitus, together with insulin resistance, has been linked to changes in circulating levels of sortilin as well as polymorphisms of sortilin 1–related VPS10-containing receptor 1 (SORC1) [20,21]. For instance, Demir et al. reported low levels of serum sortilin, and a negative correlation between sortilin and insulin resistance in 75 type 2 diabetes mellitus patients who were newly diagnosed [20]. This is the opposite of what Oh et al. reported—thatserum levels of sortilin were raised in an population of statin naive diabetic patients with coronary artery disease. They also showed that the most sortilin-elevated persons had the most HbA1c and the highest glucose levels [21].

According to earlier research, obesity, insulin resistance, and pancreatic beta-cell malfunction are some of the metabolic diseases related to PCOS [22]. However, writing regarding insulin resistance and PCOS is robust, and there are no studies looking at serum sortilin levels and its relationship with metabolic abnormalities among women with PCOS. SORC1 polymorphisms have been reported from 50 obese PCOS patients in Hrovat et al. to have no association with basal glucose, insulin, or HOMA-IR values [23]. They, however, reported that the SORC1 rs1416406 polymorphism was present, as glucose and insulin levels increased after a 120-min oral glucose tolerance test (OGTT).

Based on our findings, sortilin does not alter the insulin secretion pattern of PCOS patients when stimulated by glucose, as there were no significant correlations between sortilin levels and either glucose or insulin levels when measured at the first or second time points during the 120 min OGTT test or A1c baselines. There were statistically nonsignificant positive correlations between basal glucose and sortilin levels (*p* = 0.068). These results indicate that sortilin-induced molecular mechanisms in regulating blood glucose homeostasis may play a role in the pathogenesis of PCOS. Yet unexplained sortilin was not seen in relation to our PCOS patients and their HOMA-IR values despite the noted information on insulin resistance.

Sortilin likewise participates in lipid metabolism, specifically in the circulation of LDL-C. Genomic-wide linkage scans pinpointed the SORT1 gene to be a major determinant of LDL metabolism and of raised relative risk for CAD, owing to its location on the chromosome 1p13.3 [24]. It is possible that sortilin facilitates the uptake of LDL by macrophages, which then becomes foam cells, and this leads to platelet activation and atherosclerosis [25]. Membrane milled sortilin serves as a contractive endocytic receptor for the circulating LDL that participates in the uptake of LDL-C into the liver cells by means of LDL-receptor-independent pathways [26].

Although studies such as Dapas and Dunaif have highlighted the genomic underpinnings of PCOS, our findings suggest that sortilin does not significantly alter lipid metabolism in PCOS patients, as evidenced by the lack of a strong correlation between sortilin and lipid parameters beyond LDL-C [27]. The lysosomal degradation of ApoB100 is promoted by hepatic sortilin, which also promotes the elimination of VLDL. These actions have the effect of lowering both plasma cholesterol and triglyceride concentrations, though the literature appears to be contradictory on this [20]. For instance, fishing sortilin has been shown to alter not only the export of ApoB100 but also the number of circulating triglycerides and the level of circulating LDL-C in serum [24].

On the other hand, in their murine studies, Kjolby et al. showed that the absence of sortilin reduced the secretion of VLDL from the liver and enhanced the prospect of hypercholesterolemia and atherosclerotic lesions [28]. Also, they demonstrated that the previous hepatic overexpression of sortilin promoted lipoprotein secretion, while serum LDL levels increased. This aligns with findings by Siddiqui et al., who emphasized the genetic and metabolic complexity of PCOS, suggesting that sortilin’s role in lipid metabolism may be more intricate than previously understood [29].

According to Ogawa et al., serum sortilin levels were more raised in patients at high risk of cardiovascular disease without a history of coronary heart disease (CAD) than patients with CAD or previously healthy patients. They also found that serum sortilin levels correlated positively with LDL-C and serum triglycerides. Likewise, Demir et al. reported that serum sortilin levels decreased significantly in patients with recently diagnosed type 2 diabetes, correlating negatively with levels of LDL-C, triglycerides, and total cholesterol but positively with HDL-C levels [20].

In our research, significant inverse correlations were observed between sortilin and LDL-C levels in both PCOS patients and controls. These findings suggest that sortilin may play a role in lipid regulation in PCOS, yet its function appears to be distinct from the mechanisms observed in other metabolic conditions, warranting further investigation. The low correlations noted between lipid parameters and sortilin may be due to the variability of the study subjects and the small number of subjects involved. It is important to emphasize that women who were on treatments to modify glucose and lipid metabolism such as metformin and statins were not included to avoid disturbing the effect of these treatments.

## 5. Conclusions

This was the first study to report serum sortilin levels in PCOS patients. Our study demonstrated that sortilin may be associated with the pathogenesis of PCOS, and serum sortilin levels could have promise as a biomarker for PCOS-related metabolic dysfunction. However, the interpretation of sortilin’s role should be made cautiously due to potential confounders, sample heterogeneity, and study limitations. Longitudinal and multicenter studies are needed to establish causative relationships and confirm therapeutic potentials. Further studies with larger sample sizes are needed to validate the impact of sortilin on lipid and glucose metabolism in PCOS in order to be able to accurately assess its use as a potential therapeutic target for the management of the metabolic consequences of PCOS.

## Figures and Tables

**Table 1:** The demographic and clinical features of participants.

Variables	PCOS (*n* = 80)	Controls (*n* = 80)	*p* Value
Age, years	30.05 ± 6.85	30.12 ± 6.49	0.945
BMI, kg/m^2^	26.30 ± 4.50	26.65 ± 4.43	0.623
Waist circumference, cm	93.39 ± 12.45	92.44 ± 13.67	0.648
SBP, mmHg	108.60 ± 12.62	107.28 ± 11.57	0.493
DBP, mmHg	73.98 ± 6.51	73.54 ± 5.71	0.654
Ferriman–Gallwey score	14.77 ± 2.84	4.32 ± 1.19	<0.001 *
Glucose, mg/dL	83.97 ± 7.90	81.41 ± 5.65	0.020 *
Glucose, at 120 min with OGTT	122.90 ± 14.45	120.82 ± 11.54	0.317
HbA1c, %	5.28 ± 0.17	5.24 ± 0.18	0.199
Insulin, µIU/mL	17.41 ± 6.38	11.04 ± 4.56	<0.001 *
HOMA-IR	3.63 ± 1.43	2.21± 0.90	<0.001 *
Total cholesterol, mg/dL	204.73 ± 33.26	202.76 ± 43.47	0.747
LDL-C, mg/dL	134.45 ± 28.34	131.52 ± 27.44	0.508
HDL-C, mg/dL	42.05 ± 9.46	49.16 ± 10.80	<0.001 *
Triglyceride, mg/dL	141.14 ± 32.58	110.38 ± 29.98	<0.001 *
Hs-CRP, mg/L	1.19 ± 0.54	0.68± 0.21	<0.001 *
FSH, mIU/mL	6.75 ± 1.84	7.31 ± 1.92	0.068
LH, mIU/mL	14.03 ± 4.13	8.59 ± 3.01	<0.001 *
Estradiol, pg/mL	50.04 ± 11.98	49.26 ± 8.12	0.632
Total testosterone, nmol/L	2.88 ± 0.41	1.70 ± 0.35	<0.001 *
SHBG, nmol/L	36.99 ± 11.51	68.74 ± 14.84	<0.001 *
FAI, %	8.25 ± 1.69	2.48 ± 0.10	<0.001 *
DHEA-S, µg/dL	180.77 ± 72.09	154.06 ± 38.61	0.004 *
Sortilin, pg/mL	69.51 ± 27.75	48.60 ± 21.20	<0.001 *

PCOS: polycystic ovary syndrome; BMI: body mass index; DBP: diastolic blood pressure; SBP: systolic blood pressure; OGTT: oral glucose tolerance test; HbA1c: glycosylated hemoglobin; HOMA-IR: homeostasis model assessment of insulin resistance; LDL-C: low-density lipoprotein cholesterol; HDL-C: high-density lipoprotein cholesterol; Hs-CRP: high sensitivity C-reactive protein; FSH: follicle-stimulating hormone LH: luteinizing hormone; SHBG: sex hormone-binding globulin; FAI: free androgen index; and DHEA-S: dehydroepiandrosterone sulfate. Data were given as mean ± standard deviation. Independent samples t-test was used for comparison. A *p* value of <0.05 was considered significant (*).

**Table 2:** Correlation analyses between sortilin levels and participants’ clinical and biochemical characteristics.

	Sortilin			
	PCOS		Control	
	r	*p*	r	*p*
Age	0.054	0.173	0.035	0.102
BMI	0.074	0.216	0.104	0.189
Waist circumference	0.097	0.105	0.115	0.124
SBP	0.032	0.258	0.053	0.296
DBP	0.102	0.342	0.098	0.112
Insulin	0.131	0.097	0.084	0.103
Glucose	0.147	0.068	0.102	0.098
Glucose at 120 min with OGTT	0.034	0.105	0.054	0.201
HOMA-IR	0.135	0.094	0.093	0.102
HbA1c	0.116	0.174	0.105	0.213
FAI	0.189	0.245	0.141	0.314
Hs-CRP	0.094	0.214	0.052	0.321
Total cholesterol	−0.114	0.054	0.105	0.066
LDL-C	−0.198	0.028 *	−0.182	0.033 *
HDL-C	0.102	0.069	0.116	0.087
Triglyceride	−0.139	0.077	0.093	0.086

BMI: body mass index; SBP: systolic blood pressure; DBP: diastolic blood pressure; OGTT: oral glucose tolerance test; FAI: free androgen index; HOMA-IR: homeostasis model assessment of insulin resistance; HbA1c: glycosylated hemoglobin; FAI: free androgen index; Hs-CRP: high sensitivity C-reactive protein; HDL-C: high-density lipoprotein cholesterol; and LDL-C: low-density lipoprotein cholesterol. Pearson’s correlation analysis was used. r: Pearson’s correlation coefficient. A *p* value of <0.05 was considered significant (*).

## Data Availability

The original contributions presented in this study are included in the article. Further inquiries can be directed to the corresponding author.
